# COVID-19 infection and Hong Kong elementary children’s Chinese and English reading proficiencies: Parent-reported proficiency versus performance-based reading tests

**DOI:** 10.1371/journal.pone.0359793

**Published:** 2026-10-09

**Authors:** Hay Mar Myat Kyaw, Yixun Li, Derwin King Chung Chan, Catherine Mamaid Capio, Winnie Wan Yee Tso

**Affiliations:** 1 Department of Early Childhood Education, The Education University of Hong Kong, Hong Kong, China; 2 Department of Social Sciences, Hong Kong Metropolitan University, Hong Kong, China; 3 Department of Physiotherapy, Hong Kong Metropolitan University, Hong Kong, China; 4 Department of Pediatrics and Adolescent Medicine, The University of Hong Kong, Hong Kong, China; Southwest University, CHINA

## Abstract

Previous studies investigated the effect of the COVID-19 pandemic on children’s language development. This study explored the association between different COVID-19 infection statuses (children with no history of infection, children with a history of infection, and children with long COVID symptoms) and Hong Kong elementary children’s Chinese and English reading performance. Participants were 195 dyads of parents and elementary children from Grades 1–6 (*M*_age_ = 8;8, *SD*_age_ = 1;7, 89 boys and 106 girls) in 2022−2023. We adopted a 3 x 3 between-participant design. The two independent variables were COVID-19 status and grade levels. The dependent variables were parents’ ratings of reading proficiencies, reading proficiencies in Chinese and English, and the differences between the parents’ ratings and reading proficiencies. All measures (the language experience and proficiency questionnaire for parents’ ratings, Chinese word reading battery, and the Wechsler Individual Achievement Test – Third Edition) were adapted from published works and showed high reliability in our sample. Using Multivariate Analysis of Covariates (MANCOVA) and Analyses of covariance (ANCOVA), we examined the links of infection with covariates, including parents’ education level, number of children in the family, and monthly household income. We found that long COVID status was associated with lower parent-rated Chinese reading in the upper grades and lower Chinese spelling performance. Grade-specific differences in parent–performance discrepancy scores were also observed. These findings highlight the need for further longitudinal investigations and needs-based monitoring using both caregiver reports and performance-based assessments for children experiencing persistent health and learning difficulties.

Recent studies have shown that people with COVID-19 suffer from poorer cognitive functioning than those without, both during the infection phase as well as after being infected [1,2]. COVID-19 has also been shown to affect the development and achievement of young children: they lose time in education, as well as with their caregivers, and they suffer increased social isolation, family poverty, and stress. Altogether, these losses may affect their entire life, and may even affect their own children in the future [3]. So how, and to what extent, does COVID-19 affect language learning in young children, particularly reading? Young children are especially sensitive to environmental exposure for their language development and were seriously affected by reduced social interactions during the pandemic, as well as the closure of schools and kindergartens [4,5]. Many investigations about first language (L1) learning in primary students during the pandemic, for example, in English (United States) [6] and Dutch (the Netherlands) [7], indicated a decline in reading achievement and word reading from 2020 to 2021. However, no learning deficits in Swedish primary school children in terms of their word decoding and reading comprehension scores in Swedish (L1) were indicated during the pandemic in comparison to different cohorts (from 2017–2021) [8], as Sweden was one of the few countries that decided to keep schools open, starting from lower secondary level to preschools during the pandemic.

Most studies compared children’s test scores before and during the pandemic to explore the changes in their language development. However, simultaneous comparisons of reading achievement across different children groups, such as with or without COVID-19 infection and long COVID symptoms, were rarely observed in investigating the trends in language proficiencies. The lack of such comparisons led to the fact that differences observed in these studies are likely to be accounted for by the pandemic measures in education settings, such as school closure, face masks, and social distancing, rather than by COVID-19 infection. Hence, comparing the reading development of different groups with COVID-19 statuses in a similar context with pandemic measures would be ideal for evaluating the associations of infection to the development. Therefore, the present work seeks to fill this gap by exploring the associations of COVID-19 on Hong Kong elementary children’s language proficiencies, focusing on reading, in both first language (L1) and second language (L2) through the group comparison with different infection status (with and without COVID-19 infection, long COVID symptoms), since it was one of the places where the strictest pandemic measures were imposed during the pandemic.

## COVID infection and reading development in children

Long COVID has gained attention in the medical research field [9,10], but it has yet to be examined in educational research. There are different definitions of long COVID in health-related research. The term “long COVID” refers to the condition of having persistent symptoms (e.g., fatigue, cough, muscle aches, headache that could not be attributed to any other cause) beyond two weeks (mild disease), beyond four weeks (moderate to severe illness), and six weeks (critical patients) [9]. Consequently, many long COVID patients have reported “brain fog,” including deficits in attention, executive functioning, language, processing speed, and memory, along with increased anxiety, depression, sleep disorder, and fatigue [11]. Despite being less severe in children than in adults, concerns about the potential long-term impacts of long COVID have been raised among parents [10]. In our study, we developed an operational definition of Long COVID for our study which refers to a condition in which individuals, including children, experience persistent symptoms including fatigue, headache, cognitive deficits (brain fog), anxiety, depression, and sleep disturbances that continue after recovering from COVID-19 [9,11,12].

Existing research on the pandemic’s impact on children’s reading and language skills presents mixed findings. Some studies in Australia and Germany report no negative effects or even positive impacts on L1 reading, potentially due to shorter school closures and effective educational recovery measures [13,14]. Conversely, most research indicates declines in language proficiency, especially in disadvantaged populations, as seen in Belgium [15] and Finland [16]. However, these studies primarily examine the effects of school closures and pandemic-related disruptions, without isolating the specific impact of COVID-19 infection or long COVID symptoms on reading development.

A significant gap remains regarding whether children with a history of COVID-19 infection, particularly those experiencing long COVID symptoms, demonstrate different reading trajectories compared to children without infection. This is especially relevant given evidence of cognitive deficits, such as attention and memory problems, associated with COVID-19 [1,3], which are critical for reading acquisition and development.

After reviewing studies from other countries and regions, it is important to consider Hong Kong’s distinctive educational landscape and pandemic response measures, which set it apart from the contexts discussed earlier. Hong Kong implemented prolonged school closures and strict social distancing policies, resulting in significant disruptions to face-to-face instruction [17]. While many regions have experienced shorter closures or different mitigation strategies, Hong Kong’s rigorous approach and extended periods of remote learning have created a unique environment for examining the pandemic’s impact on children’s development. Furthermore, despite the easing of restrictions and the return to in-person classes, the long-term effects of COVID-19 infection, especially long COVID symptoms, may still influence students’ cognitive and reading abilities. Given Hong Kong’s highly competitive education system and emphasis on literacy skills, understanding how COVID-19, including persistent symptoms, affects children’s reading development is particularly relevant. This context underscores the importance of our study in filling the gap in local research and highlights why investigating COVID’s impact on Hong Kong children’s literacy outcomes is both timely and necessary to inform targeted educational support.

From a theoretical perspective, children’s reading development is often conceptualized as progressing through stages [18], and draws on cognitive functions such as attention, executive functioning, and working memory. In Chinese, a morphosyllabic writing system, visual-spatial attention and working memory contribute to orthographic processing by helping readers analyze complex character configurations, map them to meaning, and retrieve representations from long-term memory during character recognition and spelling [19,20]. By contrast, attentional and working memory resources are primarily allocated to sequential orthographic and phonological processing in English word reading and spelling [21]. Bilingual learners may additionally recruit executive functions to manage cross-linguistic competition [22]. Previous research has associated long COVID with difficulties in attention, executive functioning, and working memory [23,24]. These cognitive functions may be relevant to reading performance, particularly in the upper elementary grades (Grades 5–6), when children encounter more visually complex Chinese characters and more multisyllabic and morphologically complex English words. Retrieving and spelling increasingly complex words may place greater demands on attention, working memory, and executive control. Accordingly, long COVID-related difficulties in these cognitive domains could be associated with reading outcomes when task demands are high. This possibility provides a theoretical rationale for examining whether patterns of reading performance vary across COVID-19 status and grade level.

However, current literature lacks empirical evidence on how COVID-19 infection and long COVID symptoms influence reading development (L1 and L2) across different elementary grade levels. Addressing this gap is crucial for understanding the long-term educational impacts of COVID-19 and for developing effective interventions aimed at supporting children’s literacy in the aftermath of the pandemic.

## Potential (In)consistencies between parents’ perception and children’s reading performance

Parents’ perceptions of their children’s development provide valuable information, particularly when access to formal assessment is limited. Therefore, parent-reported measures have been widely used to assess children’s language development and cognitive functioning [25,26]. However, their correspondence with performance-based measures varies across samples and contexts [27,28]. The Realistic Accuracy Model explains that judgment accuracy depends on whether relevant behavioral cues are available to and detected by parents, and whether those cues are interpreted appropriately [29]. Applied to reading development, parents may draw on everyday observations of homework, reading behavior, school feedback, and apparent effort, whereas performance-based assessments measure specific skills under structured conditions. Thus, parental ratings and test performance may provide complementary but not identical information. This comparison is important because parental judgments may shape expectations, help-seeking, and the educational support children receive at home and through schools.

Since the COVID-19 pandemic, parents’ ratings have been increasingly utilized to explore the links between parental factors, such as involvement, stress, and burnout, and children’s academic achievement [30]. Lockdowns and school closures have intensified parent-child interactions, often amplifying parental stress, especially among families facing economic hardships or with children having special needs [31,32]. Despite the widespread use of parent perceptions during this period, few studies have directly examined how COVID-19 infection status might influence parents’ evaluations of their children’s language and reading development.

To date, only one study from the UK investigated the impact of restricted access to early childhood facilities during COVID-19 on children’s language development, relying solely on parent ratings [33]. While this study found negative effects, it did not include performance-based reading assessments of children’s performance, raising concerns about potential recall bias and the accuracy of parental reports. Recognizing this gap, our study aims to compare parents’ ratings of their children’s reading proficiency with actual test performance. This dual approach will help clarify whether parental perceptions align with children’s true abilities and how the pandemic may affect this relationship.

Furthermore, in the Chinese cultural context, collectivism emphasizes close parent-child relationships and mutual attention, which may influence parental evaluations. Given the stressors associated with COVID-19 infection and the heightened role of parents during this period, it is both timely and necessary to explore how infection status affects parental perceptions of children’s language development. Incorporating both subjective ratings and performance-based reading assessments allows us to better understand how the pandemic shapes parental evaluations and to identify potential discrepancies that could impact support strategies for children’s learning. Examining discrepancies between these two measures is also critical as parental judgments may shape help-seeking, expectations, and the kinds of support children receive at home and in collaboration with schools. Understanding these dynamics can inform targeted strategies to support children’s learning effectively during and beyond the pandemic.

## The present study

The present work aimed to investigate the associations of different COVID-19 infection statuses (children with and without a history of COVID-19 infection, and children with long COVID symptoms) to Hong Kong children’s reading performance, including both L1 (Chinese) and L2 (English). Recent studies we discussed above have shown that the COVID-19 pandemic has negatively impacted children’s developmental outcomes, including language and literacy skills, due to disruptions in learning environments. These effects can be understood through Bronfenbrenner’s Ecological Systems Theory (1979) [34], which highlights how multiple environmental layers—family, community, and societal systems—interact to influence child development and how the child-related factors shape the parent, family and the community. In our study, building on this framework, we explore how children’s COVID-19 infection status is associated with both perceived and actual reading performance in L1 and L2 during the pandemic and the differences between them. The research questions guiding the present work are:

**Research question 1**: Are there any significant differences in parent-reported reading performance (L1 and L2) among children with different COVID-19 infection status?

Based on Ecological Systems Theory, we hypothesize that there will be significant differences in parent-reported reading profiles among children with different COVID-19 infection statuses, in both L1 and L2. Specifically, parents of children experiencing long COVID are expected to rate their children’s reading abilities significantly lower than parents of children who had COVID-19 but recovered without long-term symptoms, as well as parents of children who never contracted COVID-19. This is because children with long COVID may experience ongoing symptoms and increased stress, which can negatively impact their development. Additionally, parents of children who had COVID-19 are expected to rate their children’s reading abilities lower than parents of children who never had COVID-19, since only the former group was directly affected by the illness.

**Research question 2**: Are there any significant differences in children’s reading outcomes (L1 and L2) on Performance-based reading tests according to their COVID-19 infection status?

In line with our hypotheses for RQ1 and supported by previous research indicating that COVID-19 infections can negatively impact cognitive functioning and reading skills, we expect to observe significant differences in children’s reading performance in both L1 and L2 based on their COVID-19 infection status. Ecological Systems Theory emphasizes that a child’s development is influenced by multiple interconnected systems, including health and family environments. Therefore, children experiencing long COVID symptoms may face ongoing challenges within their microsystem, such as health-related stress and reduced support, leading to lower reading outcomes compared to children who had COVID-19 but recovered without long-term symptoms, as well as those who never contracted the virus. Similarly, children who had COVID-19 are anticipated to show lower reading performance than children who never had COVID-19, reflecting the direct impact of the illness within their immediate environment and development context.

**Research question 3**: Are there any significant differences between parent-reported reading proficiencies (L1 and L2) and their children’s reading outcomes on objective proficiency tests?

Based on Ecological Systems Theory, we hypothesize that there will be significant discrepancies between parent-rated reading proficiencies in L1 and L2 and the children’s actual reading outcomes, and that these discrepancies will vary according to COVID-19 infection status. Specifically, children with COVID-19, particularly those experiencing long COVID, may have parent evaluations that differ more markedly from their actual performance, due to factors such as the potential negative impact of long COVID on their cognitive and language development, as well as reduced parent–child interactions caused by health-related restrictions. Consequently, parents of children who had COVID-19 are expected to over- or underestimate their children’s abilities more than parents of children who never contracted the virus, reflecting the influence of health status and environmental factors within the child’s immediate ecological context.

## Method

### Participants

Our target samples were elementary students and their parents in Hong Kong, with varying COVID-19 statuses, owing to our focus on the fundamental reading skills. We recruited our participants through the survey data from a larger-scale project, which investigated the prevalence of COVID-19 infection in Hong Kong children across school levels (kindergarten, elementary, and secondary school students). In the large project, participants were recruited through social media and messaging platforms, such as WhatsApp, WeChat, and several major parent forums, from October 2022 to May 2023. The inclusion criteria for the parent participants were: (1) having children aged 3–18 without any known language-related deficiency (e.g., dyslexia); (2) being residents of Hong Kong (3) being able to read and write Chinese, and (4) speaking Cantonese or Mandarin as a home language. A total of 1,244 participants (parents) out of 1,542 eligible participants were recruited by using stratified sampling to ensure a representative sample across variables, including COVID-19 infection status, children’s age groups (Kindergarten to Grade 12), gender, and residential regions across the 18 administrative districts of Hong Kong. All participating parents filled out a questionnaire that took about 20 minutes, answering questions including their children’s COVID infection status and symptoms; this survey data helped us identify potential participants for the present study. Upon institutional ethical approval, we sent invitations to eligible participants (547 parents of elementary students out of the 1,244) from the aforementioned survey study.

Due to the pandemic measures in Hong Kong, we recruited those who agreed to participate in the present study by replying to our invitations across different digital platforms. With written parental consent, our final participants were 195 dyads of parents and elementary children from Grades 1–6 (*M*_age_ = 8;8, *SD*_age_ = 1;7, 89 boys and 106 girls) in the year 2022−2023: (1) with a history of COVID-19 infection and reporting long COVID symptoms (*N* = 62), (2) with a history of COVID-19 infection but without reporting long COVID symptoms (*N* = 76), or (3) without a history of COVID-19 infection (*N* = 57) (for classifying the varying infection status: see details in the Measures section). Among 195 children participants, 83 (36 boys and 47 girls) were lower elementary students (Grades 1 and 2), 74 (34 boys and 40 girls) were students from middle grades (Grades 3 and 4), and 38 (25 boys and 13 girls) were students from upper elementary grades (Grades 5 and 6). Sample distribution is unbalanced across the nine subgroups derived from combining COVID-19 infection status and grade level. Participant counts ranged from a minimum of 9 (upper elementary students with long COVID symptoms) to a maximum of 32 (lower elementary students with a history of COVID-19). Precise sample sizes for each subgroup are detailed in Tables 1 and 2.

**Table 1:** Descriptive statistics of variables with COVID-19 status and grade levels.

	Elementary grade levels	No COVID *M (SD)*	COVID-19 *M (SD)*	Long COVID *M (SD)*	Min.	Max.
Chinese (L1)						
Parent-reported reading (10)	Lower grades (*N* = 25, 32, 26)	5.76 (2.26)	5.22 (2.30)	5.35 (2.37)	1	10
	Middle grades (*N* = 19, 28, 27)	5.42 (2.59)	6.43 (2.20)	5.56 (1.70)	1	10
	Upper grades (*N* = 13, 16, 9)	7.85 (1.73)	5.75 (1.98)	3.56 (2.30)	0	10
Character naming (150)	Lower grades (*N* = 25, 32, 26)	38.00 (24.05)	34.34 (23.40)	29.77 (12.40)	0	113
	Middle grades (*N* = 19, 28, 27)	68.94 (25.99)	68.08 (23.20)	66.16 (22.65)	9	118
	Upper grades (*N* = 13, 16, 9)	98.54 (20.28)	84.00 (29.84)	91.67 (8.06)	15	128
Character reading fluency^*^(200)	Lower grades (*N* = 25, 32, 26)	65.08 (27.05)	65.59 (19.97)	67.00 (20.12)	7	113
	Middle grades (*N* = 19, 28, 27)	84.16 (19.99)	90.90 (15.36)	89.05 (17.25)	30	119
	Upper grades (*N* = 13, 16, 9)	102.31 (15.06)	100.7(19.39)	105.50 (16.73)	60	130
Spelling (20)	Lower grades (*N* = 25, 32, 26)	2.48 (1.94)	2.52 (1.54)	1.83 (1.30)	0	8
	Middle grades (*N* = 19, 28, 27)	7.35 (4.71)	5.51 (3.70)	5.15 (3.96)	0	18
	Upper grades (*N* = 13, 16, 9)	10.40 (3.48)	7.51 (5.28)	8.77 (2.78)	0	19
English (L2)						
Parent-reported reading (10)	Lower grades (*N* = 25, 32, 26)	5.12 (2.11)	5.09 (2.49)	4.54 (2.58)	1	9
	Middle grades (*N* = 19, 28, 27)	4.28 (2.02)	5.43 (2.62)	4.37 (1.90)	1	10
	Upper grades (*N* = 13, 16, 9)	5.77 (2.86)	4.50 (2.56)	3.89 (1.69)	1	10
English word reading^*^ (75)	Lower grades (*N* = 25, 32, 26)	12.97 (9.76)	15.45 (12.65)	10.57 (8.88)	0	41
	Middle grades (*N* = 19, 28, 27)	16.42 (8.83)	18.15 (10.52)	17.20 (9.94)	2	43
	Upper grades (*N* = 13, 16, 9)	24.85 (13.07)	21.44 (10.97)	23.07 (8.70)	2	50
Decoding efficiency^*^ (52)	Lower grades (*N* = 25, 32, 26)	7.44 (6.83)	9.62 (9.37)	5.58 (5.39)	0	35
	Middle grades (*N* = 19, 28, 27)	8.21 (7.34)	11.53 (7.78)	8.71 (6.67)	1	31
	Upper grades (*N* = 13, 16, 9)	15.39 (9.20)	10.33 (8.35)	11.13 (4.33)	2	36
Spelling (45)	Lower grades (*N* = 25, 32, 26)	5.80 (6.47)	7.07 (6.01)	4.31 (3.60)	0	25
	Middle grades (*N* = 19, 28, 27)	10.22 (4.66)	10.17 (8.33)	9.25 (5.24)	0	36
	Upper grades (*N* = 13, 16, 9)	16.22 (11.35)	12.25 (8.54)	12.63 (5.11)	1	37

*Note*. ^*^ *shows the timed tasks without any maximum score possible as children can get higher scores if they can finish reading all the words before the allowance time is up. N*s *in the parentheses show the number of children in each infection status group: No COVID, COVID-19, Long COVID. Lower grades (Grades 1–2), Middle grades (Grades 3–4), Upper grades (5–6).*

**Table 2:** Descriptive statistics of variables with COVID-19 infection status and with grade levels.

		*N*	*M*	*SD*	Min.	Max.
Chinese (L1)	COVID-19 Infection Status					
Parent-reported reading (10)	Long COVID	62	5.18	2.16	0	10
	COVID-19	76	5.78	2.24	1	10
	No COVID	57	6.12	2.43	1	10
Character naming (150)	Long COVID	62	53.66	28.17	11	104
	COVID-19	76	57.47	31.99	0	128
	No COVID	57	62.11	33.71	1	118
Character reading fluency^*^ (200)	Long COVID	62	81.08	21.34	39	128
	COVID-19	76	82.49	23.42	18	130
	No COVID	57	80.70	26.49	7	130
Spelling (20)	Long COVID	62	4.18	3.74	0	18
	COVID-19	76	4.67	3.92	0	19
	No COVID	57	5.91	4.68	0	18
English (L2)	COVID-19 Infection Status					
Parent-reported reading (10)	Long COVID	62	4.37	2.16	1	9
	COVID-19	76	5.09	2.54	1	10
	No COVID	57	4.98	2.29	1	9
English word reading^*^ (75)	Long COVID	62	13.77	9.59	0	38
	COVID-19	76	17.46	11.56	0	50
	No COVID	57	19.04	10.93	0	44
Decoding efficiency^*^ (52)	Long COVID	62	6.85	6.14	0	28
	COVID-19	76	10.50	8.55	0	36
	No COVID	57	10.75	8.03	0	32
Spelling (45)	Long COVID	62	6.71	4.95	0	20
	COVID-19	76	9.28	7.62	0	36
	No COVID	57	10.77	8.19	0	37
Chinese (L1)	Grade Levels					
Parent-reported reading (10)	Lower	83	5.42	2.29	1	10
	Middle	74	5.85	2.16	1	10
	Upper	38	5.95	2.53	0	10
Character naming (150)	Lower	83	34.01	20.83	0	113
	Middle	74	67.05	23.53	9	118
	Upper	38	90.79	23.44	15	128
Character reading fluency^*^ (200)	Lower	83	66.29	21.45	7	113
	Middle	74	88.32	17.37	30	119
	Upper	38	101.53	16.99	60	130
Spelling (20)	Lower	83	2.31	1.62	0	8
	Middle	74	5.73	4.12	0	18
	Upper	38	8.82	4.31	0	19
English (L2)	Grade Levels					
Parent-reported reading (10)	Lower	83	4.93	2.40	1	9
	Middle	74	4.74	2.25	1	10
	Upper	38	4.79	2.55	1	10
English word reading^*^ (75)	Lower	83	14.10	10.82	0	41
	Middle	74	17.05	9.91	2	43
	Upper	38	21.95	11.43	2	50
Decoding efficiency^*^ (52)	Lower	83	8.18	7.80	0	35
	Middle	74	9.64	7.44	1	31
	Upper	38	11.68	8.44	2	36
Spelling (45)	Lower	83	6.37	5.61	0	25
	Middle	74	9.55	6.54	0	36
	Upper	38	13.13	9.26	1	37

*Note*. ^*^ *shows the timed tasks without any maximum score possible as children can get higher scores if they can finish reading all the words before the allowance time is up. Lower grades (Grades 1–2), Middle grades (Grades 3–4), Upper grades (5–6).*

In our study, half of the parents (*N* = 99, 51.56%) had two children between 3 and 18 years of age, followed by those with only one child (*N* = 66, 34.38%), three children (*N* = 25, 13.02%), and four children (*N* = 2, 1.04%). The majority received secondary education (*N* = 100, 51.28%). Regarding monthly family income, most parents (*N* = 59, 30.73%) reported HK$20,001-HK$40,000, which was approximately the monthly median household salary (HK$30,000) in Hong Kong, as reported by the Census and Statistics Department (2023). The other parents in our samples were 8.85% (Less than HK$10,000), 25% (HK$10,001 – HK$20,000), 13.02% (HK$40,001 – HK$60,000), 8.33% (HK$60,001 – HK$80,000), 5.21% (HK$80,001 – HK$100,000), and 8.85% (more than HK$100,001).

### Study design and procedures

We used a 3 x 3 between-participant design. The two independent variables were children’s COVID-19 status (long COVID, COVID-19, and no COVID-19) and their grade levels (cross-sectional; lower elementary grades: Grades 1 and 2; middle elementary grades: Grades 3 and 4; upper elementary grades: Grades 5 and 6, representing different stages of reading development during elementary school). The eight dependent variables were reading proficiencies in Chinese (L1) and English (L2), those reported by the parents (reading proficiencies), and outcomes of Performance-based reading tests (word reading accuracy, word reading fluency, and spelling). In addition, demographic variables (parents’ education level, household monthly income, and number of children in the family) were included as control variables.

The information sheets explaining the details of the study and their study rights were provided to all potential participants (parents), and their signed consent was returned to the research team before completing the pre-screening questionnaire to check their eligibility for participation. Then, the research team assigned all eligible participants who fulfilled the inclusion criteria to respective groups (the long COVID group, COVID group, and no-COVID group) based on the information they provided in a self-reported survey (details in the Participants section), which took approximately 10 minutes to complete.

As a next stage for testing the reading proficiencies of the participants’ children, the research team arranged time slots, based on the parents’ preferences, for both parents and their children to be available. All the testing procedures were conducted online via Zoom because of the local pandemic measures. Note that most of our objective measures require articulation and verbal responses. Due to the implementation of various pandemic control measures, including social distancing, quarantine requirements, and restrictions on close contacts, conducting face-to-face testing with children was not feasible in Hong Kong during data collection. The Hong Kong government lifted all of these measures only in December 2022, but mask-wearing was not included in the lifted measures.

We took the following steps to ensure the validity of online testing. The same well-trained research assistant conducted the entire testing session remotely, with parents present alongside their children to facilitate the process, address any connection issues that arose, and assist with immediate tasks, such as sending photographs of the children’s writing samples after the spelling tests. All necessary writing materials were carefully mailed to participants’ homes prior to the testing sessions to ensure consistency. All assessments were conducted online in a controlled manner to ensure the integrity of the testing process. Children wore headsets to hear instructions clearly, and the tasks were timed to assess their responses accurately. The sessions were video-recorded to monitor compliance and confirm that children responded independently throughout the tests. Parental assistance was reasonably minimized through these procedures. Additionally, in our sample, many children were young, and for their safety and comfort during the tasks, the presence of a parent was necessary. Parents were requested to have an alternative communication device, such as a smartphone, on hand to enable the continuation of the test in case of any connectivity issues; however, all data collection proceeded smoothly without any connection disturbances. To ensure standardization and reduce variability, all instructions were prerecorded by a female voice in the participants’ respective home languages (Cantonese or Mandarin). The testing duration was approximately 40 minutes, with Chinese reading tests administered first, followed by English tests, and a 2–3 minute break in between for all participants. Additionally, children were allowed to take breaks whenever needed to minimize fatigue and stress, thereby promoting more accurate assessment results. These comprehensive procedures, which included mailed materials, standardized prerecorded instructions, parental support, and flexible pacing, were implemented to ensure the validity, reliability, and consistency of the remote testing process. Additionally, we employed a binary scoring system (0 for incorrect answers and 1 for correct answers), which provided a clear and objective scoring criterion, thereby minimizing subjectivity and ensuring consistent and reliable scoring. In this way, we supported the validity of the assessment without requiring interrater reliability reporting. A supermarket coupon (the value of 100 HKD/12.8 USD) was provided to each participant as an incentive for the whole testing session.

### Measures

In this section, we explained the details of all the measures we used in the present work, including the COVID-19 status questionnaire, parent-reported questionnaires, and performance-based reading tests for children reading at L1 and L2. A set of questions to categorize the children’s COVID-19 status was developed following the corresponding measures from previous studies and the type of long COVID symptoms [35,36]. In the first part, the parents were required to complete the items about their children’s history of COVID-19 diagnoses, such as the frequency, the most recent date and method of diagnosis, the variant of the virus, and vaccination status at the time of diagnosis. The second section was for the children with a history of COVID-19 infections, collecting information about whether they had any persistent long COVID symptoms diagnosed by medical professionals, the duration, and whether they were treated.

Children’s reading proficiencies in L1 and L2 were assessed in two ways: parent-reported questionnaires and performance-based reading tests. Parents were asked to rate their children’s reading profiles of Chinese (L1) and English (L2) on the language experience and proficiency questionnaire (LEAP-Q) [37]. The LEAP-Q, which is a validated tool translated into more than 40 languages, including Chinese and English (https://bilingualism.northwestern.edu/leapq/), is commonly used to assess bilingual and multilingual language performance. Participants responded on an 11-point scale ranging from 0 (no proficiency) to 10 (mastery) regarding their children’s reading proficiencies in Chinese (L1) and English (L2). The parents were also asked to fill in their demographic data in this survey, including education level, monthly household income, and total number of children in the family.

### Reading measures for Chinese (L1)

Regarding the objective measures of children’s reading proficiencies (L1), the Chinese word reading battery [38], which was developed and standardized for Hong Kong primary schoolers, was used: Character Naming and Character Reading Fluency. Before testing, we surveyed children’s home language background and the medium of instruction in their schools. Children who learn Chinese in Cantonese/Mandarin in school (medium of instruction) were tested in Cantonese/Mandarin accordingly.

#### Character naming task.

To test the character naming, the children were required to read aloud a list of 150 characters (e.g., 時, 來, and 平), selected from the Chinese language textbooks used in Grades 1–6 in Hong Kong and ordered in increasing difficulty [38]. The characters were shown in a 10 × 5 matrix (10 characters per row, 5 rows in total) on each PowerPoint slide, three slides in total, during the video testing session. One point was awarded for each character the children pronounced correctly, regarding all of the onset, rime, and tone (self-correction was considered correct). Otherwise, zero points were given. There was no time limit, and the task was terminated if the child mispronounced or skipped 20 characters consecutively. The maximum score was 150, and the task was highly reliable in the present sample (*α* = .98).

#### Character reading fluency task.

To test children’s character reading fluency, they were asked to read aloud 200 characters (e.g., 是, 了, and 中) at medium difficulty level [38]. The characters were shown in a 10 × 5 matrix on each PowerPoint slide, four slides in total during the video testing session. The time limit in the original test was one minute, and we added an extra two seconds to accommodate for the delays in the internet connection. One score was awarded for each correct answer (self-correction was considered correct), and the maximum possible score was 200. The task demonstrated good reliability in the present study (*α* = .98).

#### Spelling task.

The spelling task was developed based on a large-scale character handwriting corpus of the common characters in primary school [39]. We first arranged all of the 1,600 characters into 20 strata based on their difficulty levels, i.e., the dictation accuracy of each character reported [39]. Then, one character was sampled from each stratum, leading to a pool of 20 characters ranging in increasing difficulty in this task. The children were asked to write each character down on their answer sheet after listening to an instruction, including the name of a character, an example sentence for it, and its name for the second time. The test was discontinued after the child said they didn’t know for four consecutive times. One point was awarded for each correct character the children wrote, and the scoring was strictly based on the accurate reproduction of characters, ensuring a consistent and valid assessment of spelling ability. The full spelling test score was 20, and the test was highly reliable in the present study (Cronbach’s *α* = .92).

### Reading measures for English (L2)

Regarding English (L2) reading measures, due to a lack of standardized measures for English (L2) reading proficiencies in the Hong Kong context, subsets of the UK version of the Wechsler Individual Achievement Test – Third Edition (WIAT-III) [40]: English Word Reading; Decoding Efficiency; and Spelling were used. Scoring of all subtests was conducted according to the provided rubric.

#### English word reading task.

For the English word reading, the children were asked to read aloud 75 English words (e.g., in, to, and my) in one minute, plus two seconds to accommodate the delay in the internet connection. The maximum possible score was 75, as one score was awarded for every word the children read accurately. The task demonstrated high reliability in the present sample (Cronbach’s *α* = .97).

#### Decoding efficiency task.

The decoding efficiency test comprised 52 pseudowords (e.g., ik, ab, and fip) within one minute plus two seconds to accommodate the delay in the internet connection. Each correct decoding by the child earned one point, with a maximum score of 52. Scoring was based on the rubric for correct decoding, ensuring an objective and valid assessment of the children’s performance. Similar to other measures, the task also reached high reliability (Cronbach’s *α* = .97).

#### Spelling task.

For the spelling test, children had to write down each word (45 English words) after listening to the pronunciation of each word, an example sentence for it, and the same word for the second time. One point was awarded for each character the children wrote correctly, and the maximum score was 45. The test was stopped after the children said “no” or “don’t know” for 4 consecutive times. The task also showed high reliability here (Cronbach’s *α* = .95).

### Statistical analysis

Data were analyzed by using the IBM Statistical Package for Social Sciences (SPSS, version 29) for the Frequentist analyses. Multivariate Analyses of covariance (MANCOVA) and analyses of covariance (ANCOVA) were used to investigate three research questions with covariates, including parents’ education level, number of children in the family, and monthly household income. During the pandemic, the majority of children learned at home, and hence, home factors were considered as covariates in our study. Additionally, the selection of home factors, including parents’ education level, number of children in the family, and household income as covariates, is supported by the bioecological model of human development [41].

For each research question, outcomes were modeled as a function of COVID-19 status, grade level, and their interaction, with parent education, monthly household income, and number of children in the family entered as covariates. In general form, the model was:


$$\begin{array}{cc} Y_{i}= & \beta_{\text{0}}+\beta_{\text{1}}({\text{COVID status}}_{i})+\beta_{\text{2}}({\text{Grade level}}_{i})+\beta_{\text{3}}({\text{COVID status}}_{i}\times {\text{Grade level}}_{i})+\\ & \beta_{\text{4}}({\text{Parent education}}_{i})+\beta_{\text{5}}({\text{Monthly household income}}_{i})+\beta_{\text{6}}({\text{Number of children}\text{ in the family}}_{\;i})+\varepsilon_{i} \end{array}$$

where $Y_{i}$ denotes the outcome for participant i, $\beta_{\text{0}}$ is the intercept, $\beta_{\text{1}}$–$\beta_{\text{6}}$ are regression coefficients, and $\varepsilon_{i}$is the error term.

For *RQ*1, $Y_{i}$ comprised the two parent-reported reading outcomes (Chinese and English). For *RQ*2, $Y_{i}$ comprised the six performance-based reading outcomes. For *RQ*3, discrepancy scores were computed by standardizing parent-rated and performance-based scores and then calculating their difference; ${\text{Discrepancy}}_{i}=Z({\text{Parent}-\text{rated score}}_{i})-Z({\text{Performance}-\text{based score}}_{i})$. Moreover, we used Bayesian ANCOVAs to quantify the relative evidence for models without versus with the COVID-19-status-by-grade interaction. We report BF01, where values greater than 1 favor the null-interaction model and values less than 1 favor the corresponding interaction model [42]. Analyses were conducted using JASP (version 0.8).

## Results

Tables 1 and 2 present the descriptive statistics of reading proficiencies with their COVID-19 infection status and their grade levels in our sample. The final total sample was 195; the sample size fluctuated slightly across dependent variables (with a minimum *N* = 192) due to missing data. A series of chi-square tests of independence were conducted to examine the associations between COVID-19 status (No COVID, COVID, Long COVID) and various demographic variables. The results indicated that the number of children in the family was significantly associated with COVID-19 status, *χ²* (6, 192) = 13.426, *p* = .037, suggesting that family size varies across different COVID-19 groups. Socio-economic status also showed a significant relationship with COVID-19 status, *χ²* (4, 192) = 14.427, *p* = .006, indicating differences in socio-economic levels among the groups. Parental education was significantly associated with COVID-19 status as well, *χ²* (4, 192) = 17.076, *p* = .002. In contrast, gender was not found to be significantly associated with COVID-19 status, *χ²* (2, 195) = 2.761, *p* = .251. These results show the demographic composition in our sample, in which family size, socio-economic status, and parental education are associated with COVID-19 status, whereas gender is not. This is aligned with our inclusion of covariates (number of children in the family, household income, and parent education) in our model analyses as specified in the above section.

### Correlation analyses

Two bivariate correlational analyses were conducted for L1 (Chinese) and L2 (English) to investigate the associations between parent-rated reading proficiency and the objective test outcomes of children. For L1, statistically significant positive correlations between parents’ ratings of their children’s reading and children’s test outcomes were observed (*r*s (195) = [.29,. 34], *p* < .001). Similar patterns were observed in L2 (*r*s (195) = [. 53−. 60], *p* < .001). Table 3 presents the pairwise correlations among all variables and covariates. i.e., parents’ education, monthly household income, and number of children in the family.

**Table 3:** Correlation matrix for reading proficiencies.

	2	3	4	5	6	7	8	9	10	11
1. Parents’ education	.70^**^	−.12	.27^**^	−.02	−.03	−.02	.36^**^	.39^**^	.34^**^	.34^**^
2. Monthly household income		−.053	.30^**^	−.038	−.004	.046	.35^**^	.36^**^	.35^**^	.35^**^
3. Number of children in the family			−.19^**^	.042	.005	−.039	−.27^**^	−.17^*^	−.098	−.112
4. Parent-reported Chinese reading				.54^***^	.34^***^	.29^***^	.33^***^	.31^***^	.30^***^	.34^***^
5. Chinese character naming					.83^***^	.78^***^	.07	.39^***^	.21^***^	.47^***^
6. Chinese character naming fluency						.63^***^	.11	.41^***^	.27^***^	.42^***^
7. Chinese spelling							.10	.35^***^	.23^***^	.53^***^
8. Parent-reported English reading								.60^***^	.53^***^	.55^***^
9. English word reading									.84^***^	.84^***^
10. English decoding efficiency										.74^***^
11. English spelling										

*Note*. ^*^ *p* < .05, ^**^ *p* < .01, ^***^ *p* < .001

#### RQ1: Parent ratings of their children’s reading proficiencies in L1 and L2 across COVID-19 infection status and grade levels.

For RQ1, the dependent variables were parent-reported ratings of children’s Chinese (L1) and English (L2) reading proficiency. Thus, the findings in this subsection refer to parental evaluations, rather than children’s performance on reading tests.

We conducted a two-way Multivariate Analysis of Covariance (MANCOVA) to examine differences in parent ratings across COVID-19 infection status and grade level, controlling for parents’ education level, number of children in the family, and monthly household income. The multivariate analysis showed no significant main effect of infection status, (*F* (4, 358) = 2.325, *p* = .056; *η*_*p*_*²* = .025), and no significant main effect of grade level, (*F* (4, 358) = .905, *p* = .461; *η*_*p*_*²* = .010. However, the interaction between infection status and grade level was significant, *F* (8, 358) = 2.526, *p* = .011; *η*_*p*_*²* = .053. Because the interaction was significant, we conducted follow-up two-way ANCOVAs separately for parent-rated Chinese and English reading.

For ***parent-rated Chinese reading****,* the ANCOVA revealed a significant *main effect of infection status*, (*F* (2, 180) = 3.075, *p* = .049; *η*_*p*_*²* = .033), but pairwise comparisons across the three infection groups were not significant overall (*ps* > .063). The *main effect of grade level* was not significant, (*F* (2, 180) = 1.571, *p* = .211; *η*_*p*_*²* = .017). Importantly, the *interaction between infection status and grade level* was significant, (*F* (4, 180) = 4.346, *p* = .002; *η*_*p*_*²* = .088). Because this interaction was significant, follow-up simple-effects analyses were conducted within each grade band. These analyses indicated a significant *simple effect of infection status* among upper elementary students (*F* (2, 180) = 7.641, *p* < .001; *η*_*p*_*²* = .078), showing that parents of children with long COVID rated their children’s Chinese reading significantly lower than parents of children in the no-COVID group (*p* < .001). No significant simple effects of infection status were observed in the lower or middle grades (*p*s > .089).

For ***parent-rated English reading***, there was no *significant main effect of infection status*, *F* (2, 180) = 1.322, *p* = .269; *η*_*p*_*²* = .014, no *significant main effect of grade level*, *F* (2, 180) = .282, *p* = .754; *η*_*p*_*²* = .003, and no *significant interaction between infection status and grade level*, *F* (4, 180) = 1.522, *p* = .198; *η*_*p*_*²* = .033. Because the interaction was not significant, no grade-specific simple-effects conclusions were drawn. To further evaluate the evidence concerning this interaction, a Bayesian ANCOVA was conducted. The analysis yielded *BF*_01_ = 335.412, indicating extremely strong evidence favoring the null-interaction model over the corresponding interaction model [42].

Taken together, these findings suggest that, in upper elementary grades, parents of children with long COVID tended to rate their children’s Chinese reading proficiency lower than did parents of children without COVID-19. No comparable differences were observed in lower or middle grades, and no differences were found for parent-rated English reading. Thus, the observed effect in RQ1 concerned parental ratings of Chinese reading, particularly at upper grade levels.

#### RQ2: Children’s reading performance in L1 and L2 across COVID-19 infection status and grade levels.

For RQ2, the dependent variables were children’s performance-based reading outcomes: Chinese character naming, Chinese character reading fluency, Chinese spelling, English word reading, English decoding efficiency, and English spelling. These analyses are distinct from RQ1, which concerned parent ratings, and from RQ3, which concerns discrepancy scores between parent ratings and children’s performance.

As no significant correlations were observed between the covariates used in RQ1 and the L1 reading performance measures (character naming, character reading fluency, and spelling; see Table 3), we first conducted MANOVA and ANOVA without covariates for the Chinese outcomes. We then repeated the analyses using MANCOVA and ANCOVA to maintain consistency with the analyses for English outcomes; the pattern of results was similar. Therefore, for consistency, we report the covariate-adjusted analyses below (see details in Table 4).

**Table 4:** Two-way ANCOVA for the interaction effect between COVID-19 statuses and grade levels and for the effect of COVID-19 statuses and grade levels, controlling for parents’ education level, number of children in the family, and monthly household income.

Main effect of COVID-19 statuses Dependent variable	*df*	*F*	*p*	*η*_*p*_ *²*
Parents’ rating on Chinese reading	2, 180	3.075	.049	.033
Parents’ rating on English reading	2, 180	1.322	.269	.014
Chinese character naming	2, 180	.953	.388	.010
Chinese character reading fluency	2, 180	.343	.710	.004
Chinese spelling	2, 180	3.561	.030	.038
English word reading	2, 180	.303	.739	.003
English decoding efficiency	2, 180	1.273	.283	.014
English spelling	2, 180	1.242	.292	.014
Difference between Chinese character naming and parents’ rating	2, 180	2.332	.100	.025
Difference between Chinese character reading fluency and parents’ rating	2, 180	4.125	.018	.044
Difference between Chinese spelling and parents’ rating	2, 180	2.199	.114	.024
Difference between English word reading and parents’ rating	2, 180	.862	.424	.009
Difference between English decoding efficiency and parents’ rating	2, 180	1.993	.139	.022
Difference between English spelling and parents’ rating	2, 180	2.515	.084	.027
**Main effect of grade levels** **Dependent variable**	***df***	***F***	***p***	***η*_*p*_ *²***
Parents’ rating on Chinese reading	2, 180	1.571	.211	.017
Parents’ rating on English reading	2, 180	.282	.754	.003
Chinese character naming	2, 180	92.00	<.001	.505
Chinese character reading fluency	2, 180	51.18	<.001	.363
Chinese spelling	2, 180	53.32	<.001	.372
English word reading	2, 180	14.41	<.001	.138
English decoding efficiency	2, 180	5.724	.004	.060
English spelling	2, 180	22.49	<.001	.200
Difference between Chinese character naming and parents’ rating	2, 180	38.44	<.001	.299
Difference between Chinese character reading fluency and parents’ rating	2, 180	22.23	<.001	.198
Difference between Chinese spelling and parents’ rating	2, 180	22.39	<.001	.199
Difference between English word reading and parents’ rating	2, 180	11.25	<.001	.111
Difference between English decoding efficiency and parents’ rating	2, 180	2.388	.095	.026
Difference between English spelling and parents’ rating	2, 180	17.46	<.001	.162
**Interaction effect between COVID-19 statuses and grade levels** **Dependent variable**	***df***	***F***	***p***	***η*_*p*_ *²***
Parents’ rating on Chinese reading	4, 180	4.346	.002	.088
Parents’ rating on English reading	4, 180	1.522	.198	.033
Chinese character naming	4, 180	.595	.667	.013
Chinese character reading fluency	4, 180	.278	.892	.006
Chinese spelling	4, 180	1.193	.315	.026
English word reading	4, 180	.880	.477	.019
English decoding efficiency	4, 180	1.876	.117	.040
English spelling	4, 180	.982	.418	.021
Difference between Chinese character naming and parents’ rating	4, 180	4.097	.003	.083
Difference between Chinese character reading fluency and parents’ rating	4, 180	3.144	.016	.065
Difference between Chinese spelling and parents’ rating	4, 180	4.444	.002	.090
Difference between English word reading and parents’ rating	4, 180	.916	.456	.020
Difference between English decoding efficiency and parents’ rating	4, 180	.766	.548	.017
Difference between English spelling and parents’ rating	4, 180	.878	.478	.019

*Note*. *η*_*p*_*^2^* = .0099 (small effect); *η*_*p*_*^2^* = .0588 (medium effect); *η*_*p*_*^2^* = .1379 (large effect) [53].

A two-way MANCOVA examining the six performance-based reading outcomes showed no significant main effect of infection status, (*F* (12, 350) = 1.461, *p* = .137; *η*_*p*_*²* = .048), significant main effect of grade level, *F* (12, 350) = 13.117, *p* < .001; *η*_*p*_*²* = .353, and no significant interaction between infection status and grade level, *F* (24, 612) = 0.893, *p* = .613; *η*_*p*_*²* = .030, after controlling for parents’ education level, number of children in the family, and monthly household income. Separate two-way ANCOVAs were also conducted to examine the individual outcomes.

For the **Chinese outcomes**, there was a *significant main effect of grade level* on ***character naming***, *F* (2,180) = 92.00, *p* < .001, *η*_*p*_*²* = .505. Upper elementary students showed the highest performance, and middle elementary students significantly outperformed lower elementary students (*p*s < .001). No *significant main effect of infection status* (*p* = .39) or *interaction effect* (*p* = .67) was observed. For Chinese character naming, the Bayesian ANCOVA yielded *BF*_01_ = 138.45, indicating extremely strong evidence favoring the null-interaction model over the corresponding interaction model [42]. Similarly, for ***character reading fluency***, there was a *significant main effect of grade level*, *F* (2,180) = 51.181, *p* < .001, *η*_*p*_*²* = .363. Upper elementary students performed best, and middle elementary students outperformed lower elementary students (*p*s < .001). Again, neither the main effect of infection status (*p* = .71) nor the interaction effect (*p* = .89) was significant. For Chinese character reading fluency, the Bayesian ANCOVA yielded *BF*_01_ = 107.49, indicating extremely strong evidence favoring the null-interaction model over the corresponding interaction model [42].

For ***Chinese spelling***, there was a *significant main effect of grade level*, *F* (2,180) = 53.32, *p* < .001, *η*_*p*_*²* = .372, with upper elementary students performing best and middle elementary students outperforming lower elementary students (*p*s < .001). There was also a *significant main effect of infection status*, *F* (2,180) = 3.561, *p* = .03*, η*_*p*_*²* = .038. Pairwise comparisons showed that children with long COVID performed significantly lower than children without a history of COVID-19 (*p* = .04); other pairwise comparisons were not significant (*p*s > .103). The *interaction between infection status and grade level* was not significant (*p* = .32). Given the absence of an interaction, interpretation focused on the main effects. For Chinese spelling, the Bayesian model comparisons yielded very strong evidence favoring the null-interaction model (30 < *BF*_01_s < 100) [42].

For the **English outcomes**, there was a *significant main effect of grade level* on ***English word reading***, *F* (2,180) = 14.41, *p* < .001, *η*_*p*_*²* = .138. Upper elementary students performed best, and middle elementary students outperformed lower elementary students (*p*s < .021). No *significant main effect of infection status* (*p* = .739) or *interaction effect* (*p* = .477) was found. For ***decoding efficiency***, the *main effect of grade level* was also significant, *F* (2,180) = 5.724, *p* = .004, *η*_*p*_*²* = .06, with upper elementary students performing significantly better than lower elementary students (*p* = .003). No *significant main effect of infection status* (*p* = .283) or *interaction effect (p* = .117) was detected. For ***English spelling***, there was again a *significant main effect of grade level*, *F* (2,180) = 22.489, *p* < .001, *η*_*p*_*²* = .20, with upper elementary students performing best and middle elementary students outperforming lower elementary students (*p*s < .009). Neither the *main effect of infection status* (*p* = .292) nor the *interaction effect* (*p* = .418) was significant. As for the English reading outcomes, the Bayesian model comparisons yielded BF01 values between 30 and 100, indicating very strong evidence favoring the corresponding null-interaction models [42].

Overall, the RQ2 analyses showed that children’s performance-based reading outcomes improved with grade level, with upper elementary students generally performing the best. COVID-19 status was not statistically associated with most reading outcomes. For the tested interactions, the Bayesian analyses favored the null-interaction models over the corresponding interaction models. The main exception was *Chinese spelling*, for which children with long COVID performed lower than children in the no-COVID group.

#### RQ3: Difference between children’s performance and parents’ ratings in L1 and L2 reading across COVID-19 status and grade levels.

For RQ3, the dependent variables were discrepancy scores between children’s performance-based reading outcomes and their parents’ ratings. To make parent ratings and test scores comparable, both were first standardized as z-scores, and discrepancy scores were then calculated from these standardized values. Thus, the outcomes in this subsection do not reflect reading performance itself; rather, they indicate the degree of mismatch between parents’ evaluations and children’s observed reading performance.

The six discrepancy outcomes were: (i) the difference between Chinese character naming and parent rating, (ii) the difference between Chinese character reading fluency and parent rating, (iii) the difference between Chinese spelling and parent rating, (iv) the difference between English word reading and parent rating, (v) the difference between English decoding efficiency and parent rating, and (vi) the difference between English spelling and parent rating.

A two-way MANCOVA revealed *significant main effects of grade level*, *F* (12, 350) = 7.35, *p* < .001, *η*_*p*_*²* = .201, and *COVID-19 status*, *F* (12, 350) = 2.125, *p* = .015, *η*_*p*_*²* = .068, on these discrepancy scores, controlling for parents’ education level, number of children in the family, and monthly household income. The *interaction between infection status and grade level* was not significant at the multivariate level, *F* (24, 611) = 1.269, *p* = .177, *η*_*p*_*²* = .041. To clarify the significant multivariate main effects and to examine the individual discrepancy outcomes, separate two-way ANCOVAs were conducted for each L1 and L2 discrepancy measure.

For the ***Chinese discrepancy outcomes***, there was a *significant main effect of grade level* on the ***discrepancy between Chinese character naming and parent rating***, *F* (2, 180) = 38.439, *p* < .001, *η*_*p*_*²* = .299. The largest gap was observed in the upper elementary grades, followed by the middle grades, with the smallest gap in the lower grades (all pairwise comparisons *p*s < .001). The *main effect of infection status* was not significant (*p* = .10). However, *the interaction between infection status and grade level* was significant, *F* (4, 180) = 4.097, *p* = .003, *η*_*p*_*²* = .083. As this interaction was significant, follow-up simple-effects analyses were conducted within each grade band. These showed a *significant simple effect of infection status* in the upper elementary grades, *F* (2, 180) = 6.37, *p* = .002, *η*_*p*_*²* = .066, but not in the middle or lower grades (*p*s > .139). Specifically, among upper elementary students, the *discrepancy between parent ratings and children’s character-naming performance* was significantly larger for children with long COVID than for children with or without a history of COVID-19 (*p*s < .01); no significant difference was found between the COVID and no-COVID groups (*p* = 1.00).

A similar pattern was observed for the ***discrepancy between Chinese character reading fluency and parent rating***. There was a *significant main effect of grade level*, *F* (2, 180) = 22.213, *p* < .001, *η*_*p*_*²* = .198, with the largest gaps in the upper grades, followed by the middle grades, and the smallest gaps in the lower grades (all pairwise comparisons *p*s < .021). The *main effect of infection status* was also significant, *F* (2, 180) = 4.125, *p* = .018, *η*_*p*_*²* = .044, such that the long-COVID group showed a larger discrepancy than the no-COVID group (*p* = .018); other pairwise comparisons were not significant (*ps* > .96). In addition, the *interaction between infection status and grade level* was significant, *F* (4,180) = 3.144, *p* = .016, *η*_*p*_*²* = .065. Follow-up simple-effects analyses showed a *significant simple effect of infection status* in the upper elementary grades, *F* (2,180) = 7.375, *p* < .001, *η*_*p*_*²* = .076, but not in the middle or lower grades (*p*s > .679). Specifically, among upper elementary students, the discrepancy between parent ratings and children’s character-reading-fluency performance was significantly larger for children with long COVID than for children with or without a COVID-19 history (*p*s < .049); the COVID and no-COVID groups did not differ significantly (*p* = .214).

Likewise, for the ***discrepancy between Chinese spelling and parent ratin*g**, there was *a significant main effect of grade level*, *F* (2, 180) = 22.388, *p* < .001, *η*_*p*_*²* = .199, with the largest gaps in the upper grades, followed by the middle grades, and the smallest gaps in the lower grades (all pairwise comparisons *p*s < .008). The *main effect of infection status* was not significant (*F* (2, 180) = 2.199, *p* = .114, *η*_*p*_*^2^* = .024). However, the *interaction between infection status and grade level* was significant, *F* (4,180) = 4.444, *p* = .002, *η*_*p*_*^2^* = .090. Follow-up simple-effects analyses showed a *significant simple effect of infection status* in the upper elementary grades, *F* (2,180) = 4.268, *p* = .015, *η*_*p*_*^2^* = .045, but not in the middle or lower grades (*p*s > .856). Among upper elementary students, the ***discrepancy between parent ratings and children’s Chinese spelling performance*** was significantly larger for children with long COVID than for children with or without a history of COVID-19 (*p*s < .027); again, the COVID and no-COVID groups did not differ significantly (*p* = 1.00).

For the ***English discrepancy outcomes***, there was a *significant main effect of grade level* on the ***discrepancy between English word reading and parent rating***, *F* (2, 180) = 11.248, *p* < .001, *η*_*p*_*²* = .111. Both upper and middle elementary students showed larger gaps than lower elementary students (*p*s < .024), whereas the difference between upper and middle grades was not significant (*p* = .057). Neither the *main effect of infection status* (*p* = .424) nor the *interaction effect* (*p* = .456) was significant. The Bayesian ANCOVA yielded only anecdotal evidence favoring the null-interaction model for the discrepancy in English word reading (1 < *BF*_01_ < 3) [42].

For the ***discrepancy between English decoding efficiency and parent rating***, neither the *main effect of grade level* (*p* = .095), the *main effect of infection status* (*p* = .139), nor the *interaction* (*p* = .548) was significant. The Bayesian ANCOVA yielded *BF*_01_ = 1516.877, indicating extremely strong evidence favoring the null-interaction model for the discrepancy in English decoding efficiency [42].

For the ***discrepancy between English spelling and parent rating***, there was a *significant main effect of grade level*, *F* (2, 180) = 17.455, *p* < .001, *η*_*p*_*²* = .162, with both upper and middle elementary students showing larger gaps than lower elementary students (*p*s < .001), while the difference between upper and middle grades was not statistically significant (*p* = .071). Neither the *main effect of infection status* (*p* = .084) nor the *interaction effect* (*p* = .478) was significant. The Bayesian ANCOVA yielded only anecdotal evidence favoring the null-interaction model for the discrepancy in English spelling (1 < *BF*_01_ < 3) [42].

Overall, the RQ3 analyses indicated that the gap between children’s reading performance and their parents’ ratings tended to increase with grade level, especially in the upper elementary grades. These findings should be interpreted as differences in parent–performance alignment, not as differences in reading performance alone. In Chinese, significant interactions showed that upper-elementary children with long COVID had larger discrepancies between parent ratings and performance in character naming, reading fluency, and spelling. In English, grade level influenced the size of the discrepancies in word reading and spelling, but COVID-19 status showed little evidence of association. Bayesian evidence for the English discrepancy outcomes varied in strength: it was extremely strong in favor of the null-interaction model for decoding efficiency but only anecdotal for word reading and spelling.

## Discussion

The present work aimed to investigate the association between COVID-19 infection status and the reading performance of Hong Kong children in both Chinese L1 and English L2. After adjustment for parents’ education, monthly household income, and the number of children in the family, we found that parents of upper-elementary children in the long COVID group rated their children’s Chinese reading lower than parents of children in the no-COVID-19 group, with a medium effect size. Moreover, children in the long COVID group also obtained lower Chinese spelling scores than those in the no-COVID-19 group, although this difference had a small effect size. In addition, the long COVID group showed a more negative discrepancy between parental ratings and Chinese reading performance in the upper grades, indicating that parents’ ratings were lower relative to their children’s measured performance. Taken together, these findings suggest that persistent post-COVID symptoms may be associated with difficulties in Chinese literacy and with how parents perceive those difficulties. In the following subsections, we provide possible explanations for these key findings in the context of the literature.

### Associations of COVID-19 status with Hong Kong parents’ ratings and their children’s reading performance

We assessed children’s reading using two complementary sources: parents’ ratings and performance-based reading tests. After adjustment for parents’ education, the number of children in the family, and average monthly household income, outcome-specific analyses indicated that long COVID status was associated with lower parental ratings of Chinese reading among upper-elementary children and with lower Chinese spelling scores. The difference in parental ratings among upper-elementary children was characterized by a medium effect size, whereas the difference in Chinese spelling was characterized by a small effect size. The practical significance of the latter should be interpreted cautiously and confirmed in larger prospective studies. The present data do not support extrapolation to the number of children who might experience spelling difficulties at the population level. These findings are broadly consistent with previous research linking childhood health conditions to academic outcomes, including reading [43,44], and extend this literature by examining long COVID status in Hong Kong elementary children. Nevertheless, the modest magnitude of the Chinese spelling difference warrants cautious interpretation and further investigation in larger prospective studies.

Ecological Systems Theory provides one possible explanation for the parental-rating finding. Parents’ judgments are informed not only by academic performance but also by children’s behavior and functioning within the home environment [34]. Parents observing persistent fatigue, concentration difficulties, or behavioral changes may consequently perceive greater difficulty in their children’s reading, particularly in the upper grades, when literacy demands increase. Parental ratings may, therefore, capture aspects of children’s everyday functioning that are not fully represented by performance on a specific reading test. Direct assessment of symptoms and everyday learning behavior would help clarify this possibility in future research.

Moreover, previous studies have documented pandemic-related declines in children’s reading performance [7,45,46]. Although those studies primarily examined the broader effects of the pandemic rather than long COVID, they provide relevant context for the present Chinese spelling finding. All children in the present study experienced disruptions such as school closures, remote instruction, mask-wearing, and social isolation [47]. Therefore, the difference associated with long COVID emerged against a background of pandemic-related disruption shared by the entire sample. Persistent symptoms involving attention, memory, and executive functioning [48] provide one plausible explanation, because Chinese spelling requires the retrieval and accurate reproduction of complex orthographic representations. This cognitive account remains a hypothesis to be examined directly in the future, rather than evidence that long COVID caused the observed spelling difference.

Furthermore, no statistically significant Chinese spelling difference was detected between the COVID-19 and no-COVID-19 groups, whereas an outcome-specific difference was observed between the long COVID and no-COVID-19 groups. Within this sample, the pattern suggests that persistent symptoms may be more relevant to Chinese spelling than a history of infection without persistent symptoms. It does not, however, establish that COVID-19 infection alone is unrelated to reading. Note that the present work relied on parent-reported COVID-19 status, which may also have blurred the group comparisons if undiagnosed or asymptomatic infections were included in the no-COVID-19 group. Such misclassification could have reduced, increased, or otherwise altered the estimated differences. Future studies should move forward with examining symptom persistence and severity using prospectively verified infection information.

Lastly, we found that COVID-19 status was not significantly associated with the measured English reading outcomes or corresponding parental ratings. This finding can be interpreted through the biobehavioral systems approach [49], which conceptualizes literacy outcomes as arising from interactions between children’s cognitive characteristics and their educational environments. From this perspective, the different findings for Chinese and English may reflect differences in task demands and instructional contexts. Chinese spelling requires the retrieval and reproduction of complex orthographic representations, whereas English word reading and spelling permit greater use of sequential phonological, orthographic, and morphological cues. English literacy in Hong Kong may also receive substantial structured instruction and repeated practice. Together, these characteristics could make the English tasks less sensitive than Chinese spelling to variations in attention or working memory associated with persistent symptoms.

Alternatively, the null English findings could reflect measurement properties, language exposure, or limited statistical power within the subgroups. Because cognitive functioning and instructional exposure were not assessed in our work, these explanations remain hypotheses rather than demonstrated mechanisms. Moreover, previous reports of pandemic-related difficulties in L2 development [15,16] indicate that L2 (English in the present work) outcomes should not be considered generally protected. Therefore, our findings are consistent with the possibility that associations between health status and literacy may vary across first and second languages, task demands, and educational contexts, but not that L2 instructional support necessarily shields children from the effects of long COVID. Future studies can clarify this potential biobehavioral account by directly assessing cognitive symptoms and instructional exposure.

### Associations of COVID-19 status with discrepancies between Hong Kong parents’ ratings and children’s reading performance

Few previous studies have examined whether COVID-19 status is associated with the relationship between parents’ ratings and children’s performance-based reading outcomes. In our study, we found that among upper-elementary children, the long COVID group had more negative standardized discrepancy scores for Chinese character naming, reading fluency, and spelling than both the COVID-19 and no-COVID-19 groups. The effect sizes were medium for Chinese character naming and reading fluency, and small for Chinese spelling. The direction and composition of the discrepancy scores are important for interpreting this pattern. The scores were calculated as *z*(parental rating) minus *z*(performance); therefore, a more negative score indicates that parents rated their children’s reading lower relative to their children’s performance. In the upper-elementary long COVID group, the more negative discrepancy was driven primarily by lower parental ratings rather than a comparably large reduction in performance-based scores. Therefore, this finding represents a relative divergence between the two measures, not direct evidence that parents were inaccurate or that children’s performance had substantially deteriorated.

The Realistic Accuracy Model provides one possible explanation for this divergence [29]. According to the model, parental judgments depend on the cues available to parents, particularly observable behaviors encountered during everyday learning [29]. Parents of children with persistent symptoms may observe fatigue, difficulty sustaining attention, increased effort, or inconsistent performance during reading activities. Such observations could contribute to lower overall ratings even when a structured assessment captures only a modest performance difference. The increasing demands of Chinese reading in the upper grades may also make these everyday difficulties more noticeable. Alternatively, parents’ knowledge of their children’s persistent symptoms could shape their expectations when evaluating academic performance. These possibilities suggest that parental ratings may incorporate information about children’s daily functioning and illness context in addition to their performance on specific reading tasks.

The medium effects for character naming and reading fluency indicate that the divergence between parental ratings and performance was most pronounced for these outcomes, whereas the corresponding spelling divergence was smaller. These effects do not support extrapolation to the number of children who may require additional support at the population level. Their practical relevance instead lies in showing that parental ratings and performance-based assessments may provide different but complementary information. When the two sources diverge, educators and researchers should consider both the child’s measured skills and the broader learning behaviors observed by parents, rather than assuming that either source alone provides a complete assessment.

Regarding English L2, we did not find COVID-19-status differences in the discrepancy scores for English reading, and the associations between parental ratings and performance appeared descriptively stronger for English than for Chinese. Within the Realistic Accuracy Model, one possible explanation is that the educational and social importance of English in Hong Kong provides parents with more frequent and visible cues—such as homework, test results, and structured practice—on which to base their judgments [50,51]. This greater availability of performance-related information could facilitate closer alignment between parental ratings and measured English performance. However, the study did not directly assess parents’ monitoring practices, English instructional exposure, or the availability of such cues; measurement differences and limited subgroup power also remain possible explanations. Thus, it is too far-fetched to establish that parents are inherently more accurate evaluators of English than Chinese reading.

Overall, we argue that parental ratings should be treated as a complementary source of information rather than a substitute for performance-based assessment. Future studies could analyze parental ratings and children’s performance jointly using regression-based moderation or polynomial regression with response-surface analysis. These approaches would help distinguish changes in parental ratings from changes in children’s performance, and clarify whether their relationship varies according to symptom severity, grade level, language exposure, or instructional context.

## Limitations and future directions

Several limitations should be considered when interpreting the present findings. First, the cross-sectional design and absence of pre-pandemic baseline reading data preclude establishing temporal order or causal relationships. The observed group differences may have preceded COVID-19 infection or may partly reflect selection effects and other unmeasured differences among the groups. Nevertheless, the study compared three COVID-19-status groups assessed during the same period and within the same broader pandemic context, while adjusting for parents’ education, monthly household income, and the number of children in the family. As such, this study provides preliminary evidence about contemporaneous associations and identifies specific patterns requiring further investigation. Future studies may use longitudinal designs with pre-infection or pre-pandemic baseline measurements to determine whether changes in reading performance follow infection or the development of persistent symptoms.

Second, voluntary sampling limits the generalizability of the findings, and some grade-by-COVID-19-status subgroups were small, with the smallest analytic cell comprising nine participants. These small cell sizes reduced statistical power and increased uncertainty in both statistically significant and nonsignificant subgroup comparisons. Nevertheless, our sample enabled preliminary comparisons across three COVID-19-status groups and three grade levels, and the reporting of subgroup sizes and effect sizes allows the strength and precision of the findings to be evaluated appropriately. Future studies could recruit larger, more balanced, and preferably probability-based samples, with adequate power for planned subgroup and interaction analyses.

Third, COVID-19 status was based on caregiver reports in our work, and the reported diagnoses and persistent symptoms were not independently verified by the research team. Differences in testing, symptom recognition, and access to diagnosis may have resulted in misclassification, including the possible placement of children with undiagnosed or asymptomatic infections in the no-COVID-19 group. The direction and magnitude of any resulting bias cannot be determined because misclassification could have attenuated, exaggerated, or otherwise altered the estimated differences among the three groups. Nevertheless, we used predefined classifications informed by established medical criteria [9,11], which provide a feasible method of distinguishing reported infection with and without persistent symptoms during pandemic-related restrictions. This distinction allowed the study to examine long COVID status as a separate correlate of reading outcomes rather than treating all previous infections as equivalent. Future research should verify infection and long COVID status through medical records or prospective clinical assessment, and should document symptom type, severity, and duration.

Fourth, the English reading measures used in our work were not standardized for Hong Kong children, and all reading assessments were administered remotely in participants’ homes with parents present. These conditions may have introduced variation in the testing environment, parental involvement, attention, and motivation. Nevertheless, the English measure demonstrated high internal consistency in the present sample, and standardized administration procedures—including headset use, real-time monitoring, scheduled breaks, and video recording—were implemented to reduce external influence. These procedures support the use of the results for preliminary within-sample comparisons, although they do not establish the measure’s validity or equivalence across groups. Future studies should use standardized and locally validated measures in both languages when available and, where feasible, conduct supervised in-person assessments or employ remote protocols designed to minimize environmental and parental influence.

Finally, the study did not measure children’s educational exposure during the pandemic, including the type, frequency, and duration of Chinese and English instruction received online or in person. Differences in instructional access and learning opportunities may have contributed to variation in reading outcomes [33,52]. Nevertheless, all participants were educated within the same Hong Kong school system and experienced the broader system-wide disruptions associated with the pandemic. Examining children within this shared educational context reduced some contextual heterogeneity and allowed the study to identify associations that remained after adjustment for key family-level covariates. Therefore, our findings provide a basis for investigating how health status and educational exposure may jointly relate to literacy outcomes. Future studies should directly measure instructional format, lesson frequency, school absence, language exposure, and access to supplementary learning, and test whether these factors explain or modify associations between long COVID status and reading performance.

### Educational implications

The present findings provide a basis for a cautious, needs-based approach for schools to identifying and responding to literacy challenges among children reporting persistent post-COVID symptoms. First, schools may consider monitoring students’ literacy needs through multiple sources, including curriculum-based assessments, teacher observations, and caregiver reports. Because parental ratings and performance-based measures may capture different aspects of children’s functioning, discrepancies between them should prompt further review rather than an assumption that either source is inaccurate. Moreover, support should be based on each child’s demonstrated educational needs rather than COVID-19 status alone. Where existing assessments identify difficulty in Chinese reading or spelling, schools may provide the literacy support ordinarily available to students with comparable needs and monitor their progress over time. Furthermore, schools may facilitate communication among parents, teachers, and relevant health professionals when persistent symptoms coincide with learning concerns. Such communication may help distinguish difficulties observed across everyday settings from those detected in formal assessments. However, the effectiveness of new cognitive-screening programs, specialized spelling interventions, or structured communication protocols should be evaluated prospectively before they are recommended as measures specifically for children with long COVID.

## Conclusion

This study extends research on children’s literacy during the COVID-19 pandemic by considering reported infection status and persistent symptoms alongside both parental ratings and performance-based assessments in Chinese and English. This multi-source, bilingual approach highlights the importance of examining children’s health and literacy within their developmental, linguistic, educational, and family contexts. Our findings provide a foundation for future longitudinal research integrating verified clinical information, standardized bilingual assessments, and detailed measures of educational exposure. Such research is needed to inform targeted and evidence-based support for children experiencing persistent health and learning difficulties.
